# Rodent host population dynamics drive zoonotic Lyme Borreliosis and Orthohantavirus infections in humans in Northern Europe

**DOI:** 10.1038/s41598-021-95000-y

**Published:** 2021-08-09

**Authors:** Mahdi Aminikhah, Jukka T. Forsman, Esa Koskela, Tapio Mappes, Jussi Sane, Jukka Ollgren, Sami M. Kivelä, Eva R. Kallio

**Affiliations:** 1 Department of Ecology and Genetics, University of Oulu, PO Box 3000, 90014 Oulu, Finland; 2 Natural Resources Institute Finland (Luke), University of Oulu, Paavo Havaksen tie 3, 90014 Oulu, Finland; 3 Department of Biological and Environmental Science, University of Jyväskylä, P.O. Box 35, 40014 Jyväskylä, Finland; 4 Department of Health Security, National Institute for Health and Welfare, Helsinki, Finland

**Keywords:** Ecology, Diseases

## Abstract

Zoonotic diseases, caused by pathogens transmitted between other vertebrate animals and humans, pose a major risk to human health. Rodents are important reservoir hosts for many zoonotic pathogens, and rodent population dynamics affect the infection dynamics of rodent-borne diseases, such as diseases caused by hantaviruses. However, the role of rodent population dynamics in determining the infection dynamics of rodent-associated tick-borne diseases, such as Lyme borreliosis (LB), caused by *Borrelia burgdorferi *sensu lato bacteria, have gained limited attention in Northern Europe, despite the multiannual abundance fluctuations, the so-called vole cycles, that characterise rodent population dynamics in the region. Here, we quantify the associations between rodent abundance and LB human cases and Puumala Orthohantavirus (PUUV) infections by using two time series (25-year and 9-year) in Finland. Both bank vole (*Myodes glareolus*) abundance as well as LB and PUUV infection incidence in humans showed approximately 3-year cycles. Without vector transmitted PUUV infections followed the bank vole host abundance fluctuations with two-month time lag, whereas tick-transmitted LB was associated with bank vole abundance ca. 12 and 24 months earlier. However, the strength of association between LB incidence and bank vole abundance ca. 12 months before varied over the study years. This study highlights that the human risk to acquire rodent-borne pathogens, as well as rodent-associated tick-borne pathogens is associated with the vole cycles in Northern Fennoscandia, yet with complex time lags.

## Introduction

Zoonotic diseases—diseases caused by pathogens transmitted between non-human vertebrate animals and humans—pose a substantial health threat to humans^1–3^. While zoonotic pathogens contribute to emerging infectious diseases, such as the current pandemic of coronavirus disease (COVID-19)^4,5^, many zoonotic pathogens are persistently transmitted from wildlife hosts to humans^3,6–10^. For instance, Orthohantaviruses (genus Orthohantavirus, family *Bunyaviridae*) are rodent-borne pathogens^11^ that are transmitted from rodent hosts to humans without vectors, as inhaled aerosols^12,13^. Orthohantaviruses cause hemorrhagic fever with renal syndrome (HFRS) and hantavirus cardiopulmonary syndrome (HCPS) depending on the virus, with approximately 100,000 diagnosed infections annually across the world^14^. In Europe, the most common rodent-borne disease is nephropathia epidemica (NE), which is a mild form of HFRS, caused by Puumala orthohantavirus (PUUV)^15^. Meanwhile, many wildlife-originating zoonoses are transmitted by vectors. For example, *Borrelia burgdorferi *sensu lato (s. l.) bacteria, including rodent-associated *Borrelia afzelii*, which cause tick-borne Lyme Borreliosis (LB) disease in humans, are transmitted by *Ixodes* spp. ticks from wildlife reservoir hosts to humans^16^. It has been estimated that there have been approximately 230,000 human infections in Western Europe in recent years^17^, whereas the Centers for Disease Control and Prevention (CDC) have estimated more than approximately 300,000 new cases in the United States yearly^18^. In order to predict and reduce the risks caused by wildlife-originated zoonotic diseases, understanding the role of their reservoir host dynamics in driving human infection dynamics is crucial^19–21^.

Rodents (order Rodentia) are important reservoirs for zoonotic pathogens^7,22^. Often, rodent population densities fluctuate either seasonally or annually, which may translate into variations in human infection risk caused by rodent-borne pathogens^19^. For instance, Northern Fennoscandian vole cycles, which are multiannual abundance fluctuations of common rodents, three to five years in length, have a high amplitude, coincide with all sympatric vole species and are synchronous over large areas^23–26^, are known to affect human PUUV infection incidence^21,27–30^.

Even though rodents are well-known reservoirs of pathogens, the role of rodent population fluctuations in the epidemiology of rodent-associated tick-borne diseases remains to be clarified. Mostly food-driven rodent abundance fluctuations have been shown to influence density of nymphal ticks, *B. burgdorferi* s. l. infection prevalence in nymphs and/or the density of infected nymphs in the following year in North America^31–33^ and in Central Europe^34–37^. Meanwhile, the mostly predator-driven vole abundance fluctuations in Fennoscandia^23–26^ have received less attention in driving human tick-borne disease risk. While density of infected nymphs and nymphal infection prevalence are commonly used as the measure for human infection risk^31,33,35–37^, studies quantifying whether rodent abundance variations are translated into human disease cases are more scant. Moreover, the findings are not unequivocal with some studies showing positive but others showing no association between rodent abundance and following year human disease^38–40^. Consequently, it remains enigmatic how the circulation of rodent-associated tick-borne pathogens is maintained in over very low reservoir host densities that may last over extended time periods, which characterise the multiannually fluctuating vole populations in Northern Europe^41^. Yet, LB persists in these regions, with approximately 6000–7000 human infections annually, mainly caused by *B. afzelii* that is transmitted by generalist ticks *I. ricinus* and *I. persulcatus*^42,43^ in Finland^44^. One of the most common rodent species in Northern Europe is the bank vole (*Myodes glareolus*)^45^, which is considered as a hyperreservoir (species that carry out two or more zoonotic pathogens) of zoonotic pathogens^7^, including PUUV^46^ and tick-borne *Borrelia afzelii,* one of the agents causing LB^47,48^. An association between bank vole abundance and LB incidence is expected as *Borrelia* infection prevalence in ticks is positively correlated with bank vole abundance^35,49,50^.

Here, we aim to characterize the temporal dynamics of Lyme Borreliosis and PUUV infections in humans in relation to the abundance fluctuations of the rodent reservoir host, the bank vole, in Finland. While human PUUV infection epidemics has been shown to follow bank vole abundance fluctuations with short time lags^21,29,51^, we were specifically interested in quantifying the relationship between human LB cases and rodent abundance fluctuations. Human LB infection incidence is expected to follow bank vole abundance with a much longer time lag than human PUUV infection incidence, as *Borrelia* bacteria are transmitted from the reservoir hosts to humans by ticks^34^. The tick life-cycle necessarily introduces time lags in pathogen transmission; ticks typically acquire the pathogen in the larval stage when feeding on a reservoir host and can then transmit the pathogen to humans in subsequent life stages, either as a nymph (typically in the following year) or as an adult (typically two years later)^52,53^.

## Methods

### Ethics statement

The study was conducted in accordance with the effective national and institutional regulations and guidelines (currently Finnish Act on the Protection of Animals used for Scientific or Educational Purposes (497/2013), that follows the directive 2010/63/EU), and was licensed by the National Project Authorisation Board (ESLH-2008-04660/Ym-23, ESLH-2009-09663/Ym-23, ESAVI-3834-04.10.03/2011, ESAVI-7256-04.10.07/2014, ESAVI-3981/2018). The human infection incidence data have not included any personal information, only numbers of cases per hospital district. Therefore, no ethics permission for the use of the human data was needed*.*

### Human infection data

We used National Institute for Health and Welfare data on monthly incidences (infections per 100 000 inhabitants) of laboratory-diagnosed LB (LBlab) caused by *Borrelia* spp. infections and NE caused by PUUV infections from 1995 to the May of 2019 (data available at www.thl.fi/ttr/gen/rpt/tilastot.html), as well as data on symptom-based LB (LBsym) cases that are available from 2011 to the May of 2019 (outpatient healthcare visits from the primary healthcare units). LBsym data consist of illnesses diagnosed with the ICD-10 code A69.2 (Lyme borreliosis), which basically refers to the clinical diagnosis of erythema migrans, the ring-like rash. We used human infection data from Northern Savo (Pohjois-Savo, PS) and Central Finland (Keski-Suomi, KS) hospital districts, these hospital districts surrounding the rodent monitoring site in Central Finland (Fig. S1).

### Bank vole data

Bank vole abundance was monitored in 100 km^2^ area in Konnevesi in Central Finland (62° 34′ N, 26° 24′ E) 1995–2019 (Fig. S1). Trapping was conducted four times per year, in May, June/July, August and October/November in 1995–2019, except in 2016–2019, when the last annual trapping was not carried out. The study area consists of 20 trapping sites in habitats preferred by bank voles, each with four Ugglan multi-capture rodent traps which were set over two nights. We used the number of bank voles captured during the total of 160 trapping nights (20 locations × 4 traps × 2 nights) per trapping session as the bank vole abundance index. We used linear interpolation to estimate bank vole abundances for the non-trapped months^21^. This is expected to give a reliable estimation of bank vole abundances for non-trapped months since our trappings were assumed to have matched with the annual minimum (May) and maximum (August or October/November) sizes of local bank vole populations. In addition, these data match with the National Resources Institute Finland (LUKE) data in terms of multiannual abundance fluctuation^54^.

### De-seasonalization of data

A minimum for the incidence of LB cases and PUUV infections as well as bank vole abundance occurs in May (Fig. S2). So, we defined as a “biological year” the period from 1st of May in calendar year *t* to the end of April in calendar year *t* + 1. At the beginning of the biological year in May, bank voles start to reproduce^55^ and ticks activate after winter in the study region^56^. Because the incidence of LBlab, LBsym^44^ and PUUV infections^27^, as well as bank vole host population dynamics^55^ show strong seasonal fluctuations (Fig. S2), we removed the seasonal effect from the monthly LB and PUUV infection incidence data with the transformation^21^

1 $$d\left( y \right)_{ijk} = y_{ijk} - \overline{{y_{k} }}$$

where $d\left( y \right)_{ijk}$ is the de-seasonalized value of *y* (*y* = {LBlab incidence, LBsym incidence, PUUV incidence}) in year *j*, month $k$ and hospital district $i$ (*i* = {Northern Savo, Central Finland}). Because we had only a single bank vole abundance data point per month, de-seasonalization was done with the formula

2 $$d\left( y \right)_{jk} = y_{jk} - \overline{{y_{k} }}$$

to these data (Fig. S3). Similarly, as in Eq. 1, *j* refers to year and *k* to month in Eq. 2.

### Statistical analysis

**Figure 1:**
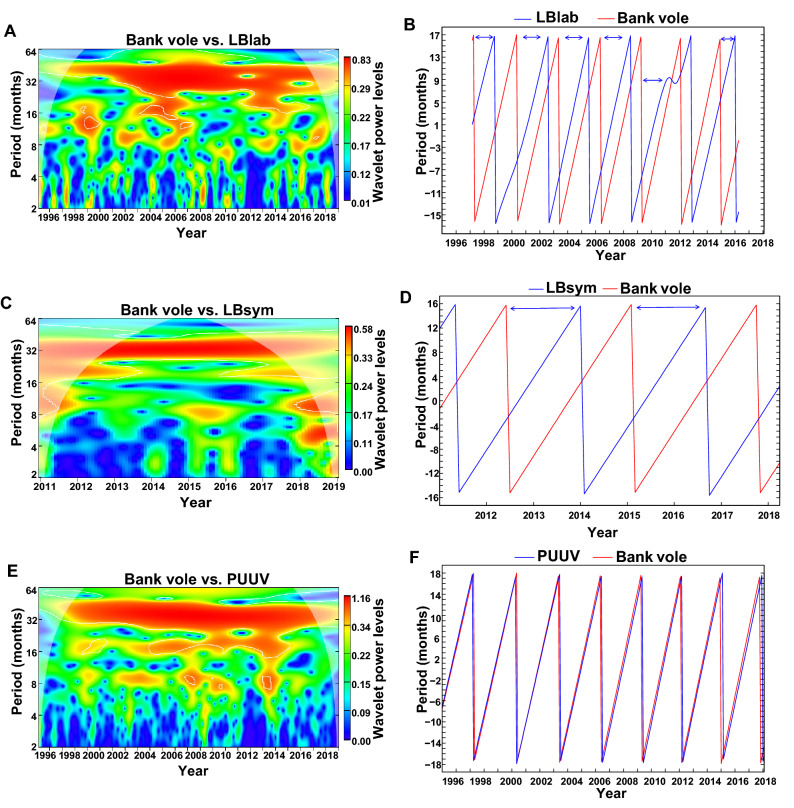
Cross-wavelet power spectra and phase-difference between human infection incidence (LB laboratory, LB symptoms, PUUV) and bank vole abundance. (**A**) Cross-wavelet power between bank vole abundance and LB laboratory incidence. (**B**) Phase-difference plot for LB laboratory incidence (blue line) and bank vole abundance (red line) for the periodicity with a wavelength of 36 months. (**C**) Cross-wavelet power between bank vole abundance and LB symptoms incidence. (**D**) Phase-difference plot for LB symptoms incidence (blue line) and bank vole abundance (red line) for the periodicity with a wavelength of 34 months. (**E**) Cross-wavelet power between bank vole abundance and PUUV incidence. (**F**) Phase-difference plot for PUUV incidence (blue line) and bank vole abundance (red line) for the periodicity with a wavelength of 36 months. In (**A**,**C**,**E**) the coherence power is presented by the color gradient from low values (blue) to high values (red). Light color at edges indicates areas influenced by edge effects. Blue arrows in panels (**B**,**D**) highlight the time lags with which LB incidence follows bank vole abundance. All the time series are de-seasonalized (see “Methods” for more details). Figure generated using R version 3.5.3^58^ software with the ‘WaveletComp’^57^ package.

To quantify the association between each of LBlab, LBsym and PUUV infection incidences and bank vole abundance variations, we used wavelet analysis as implemented in the package ‘WaveletComp’^57^ in R version 3.5.3^58^. Wavelet coherency and phase difference analyses were used to estimate the relationship between the incidence of human infections (LBlab, LBsym, PUUV infections) and bank vole abundance. The Wavelet power spectrum of two time series (human infection incidence and bank vole abundance) reveals correlated periodicity of two non-stationary time series^59^. Wavelet phase-difference reveals the time lag of correlated oscillations given a certain wavelength of periodicity. Thus, we first identified the periodicities at which human disease incidence (LBlab, LBsym, PUUV infections; all de-seasonalized) and bank vole abundance (de-seasonalized) time series are correlated with wavelet coherency analysis and, second, used the wavelet phase-difference analysis to find out the time lags between human disease incidence and bank vole abundance at those particular periodicities (Fig. 1B,D,F).

We further quantified the association between preceding bank vole abundance and human infections using linear mixed model (LMM) approach. We used linear mixed-effects models (LMMs; R function ‘lme’ from package ‘nlme’^60^) fitted with the maximum likelihood method to examine the relationship between de-seasonalized monthly infection incidence data (separately for each of LBlab, LBsym and PUUV infections) and de-seasonalized bank vole abundance data. We selected the bank vole abundance time lags, i.e. short (10 months for LBsym, 12 months for LBlab and 2 months for PUUV) and long (24 month for LBlab and LBsym) to be considered based on the results of the wavelet analysis (see above), biological knowledge of species interactions^31–33,35–37^ and earlier reports in literature^35,49,50^. The wavelet analysis suggested that, in the periodicity of 36 months, LBlab incidence follows bank vole abundance with approximately 24-month time lags and, in the periodicity of 34 months, LBsym incidence follows bank vole abundance with approximately an 18-month time lag (Fig. 1). A time lag of approximately 12 months is expected for LB because such a time lag reflects the time it takes from a larval tick that became infected by feeding an infected rodent host to develop into the nymphal stage that can infect a human. Hence, we expected a delay of approximately one year between rodent abundance and tick infection prevalence or density of infected ticks, which are predictors for human infection risk^31,33,35–37^, or between rodent abundance and human infections as reported in the literature^39,61^. Therefore, we considered a time lag of 12 months for LBlab and a time lag of 10 months for LBsym in all LMM analyses. A shorter time lag was set for LBsym than LBlab because symptom-based diagnosis of LB typically takes place soon after the tick bite, whereas laboratory-based diagnosis is delayed due to the longer time required for the development of symptoms that lead to a laboratory test of LB infection^44^. In addition to the short time lags of 12 (LBlab) and 10 (LBsym) months, we also included a time lag of 24 months for both LBlab and LBsym in subsequent analyses because the wavelet analysis suggest a 24-month lag between bank vole abundance and human LB infection time series. This is biologically motivated as human infections caused by adult ticks typically occur two years after the ticks acquired *Borrelia* spp. bacteria from a bank vole in the larval stage. We assumed a priori the shortest time lags to be the most important ones for the dynamics of the study system, so we considered interactions with the biological year effects only for the 10- and 12-month bank vole abundance time lags. We assessed these interactions because we aimed to find out whether a potential dependency of LB incidence in the human population is similarly associated with bank vole abundance through the study period. We considered this important because the amplitude of bank vole population fluctuations changed over the study period (Fig. S3). To avoid estimation problems due to autocorrelation between bank vole abundance effects associated with different time lags, we considered only the main effect of the 24-month bank vole abundance lag in subsequent analyses.

The wavelet analysis suggested that PUUV infection incidence in the human population follows bank vole abundance fluctuations with a very short time lag, which is consistent with earlier work suggesting a two-month time lag^21^. This is expected because PUUV is transmitted from bank voles to humans without any vectors and the life span of the virus is limited in the environment^62^. Thus, we considered a two-month time lag in LMM analysis of PUUV incidence data.

We used multi-model inference^63^. First, we, defined a biologically meaningful global model (i.e., the most extensive model considered) and, second, derived the set of all meaningful simpler models from the global model (function ‘dredge’ in R package ‘MuMin’^64^). Finally, we averaged over the set of models that were within two AICc (Akaike’s information criterion corrected for small sample sizes) units from the model with the lowest AICc value (function ‘model.avg’ in R package ‘MuMin’^64^).

For the global model for LBlab, we set biological year (Year; continuous variable), bank vole abundance 12 months earlier (Bank vole(lag12); continuous variable) and its interaction with biological year, bank vole abundance 24 months earlier (Bank vole(lag24); continuous variable), hospital district (2 levels) and the interaction between hospital district and biological year as fixed effects. The interaction between biological year and hospital district was included because the time series plot (Fig. S3A) suggested different temporal changes in LB incidence in the two hospital districts. Month was set as a random effect in all models for LBlab to control for the correlation of month-specific observations from the two neighboring hospital districts. To control for temporal autocorrelation of residuals, we specified an autocorrelation function by first using the ‘auto.arima’ function from R package ‘forecast’^65^. This preliminary check of the autocorrelation structure suggested an autocorrelation (AR) model with a 2-month lag. Hence, we specified an AR(2) autocorrelation for the residuals in all LMMs for LBlab. As the residual plots of the LMM indicated a missing non-linear biological year effect, we added the square of biological year and its interaction with bank vole abundance 12 months earlier in the global model. Finally, we also checked if a weather variable (North Atlantic Oscillation [NAO] index) could explain LBlab incidence variation because tick activity is known to be weather dependent^66,67^. Because no NAO effect (two-month time lag; relevant when considering weather-dependency of tick activity) was detected (Supplementary Appendix A), NAO was ignored in multi-model inference described here.

LBsym data were analysed as explained above, except that a 10-month bank vole abundance time lag was used instead of 12-month one. Residual plots of the global model for LBsym did not indicate missing non-linear effects, so there was no need to add the square of biological year or interactions including it in the model.

Variation in PUUV infection incidence was also analyzed similarly as LBlab incidence explained above, with a few exceptions. ARMA(1,1) correlation structure appeared best for residual autocorrelation and was, therefore, used in all models for PUUV infections. The only bank vole abundance time lag considered was the two-month one. There were no signs of missing non-linearities in the global model based on residual plots and second order of year.

## Results

The time series of bank vole abundance shared a periodicity of approximately 18–36 months with incidence (cases per 100,000 inhabitants per month) of LBlab (Fig. 1A), a periodicity of approximately 30–36 months with incidence of LBsym (Fig. 1C), and a periodicity of approximately 18–36 months with incidence of PUUV infection (Fig. 1E). The phase difference plots suggested rather long, approximately 18 to 24-month time lags between the bank vole abundance and LB incidences (Fig. 1B, D), whereas no time lag was clear in between the time series of bank vole and PUUV infection incidence (Fig. 1F).

**Figure 2:**
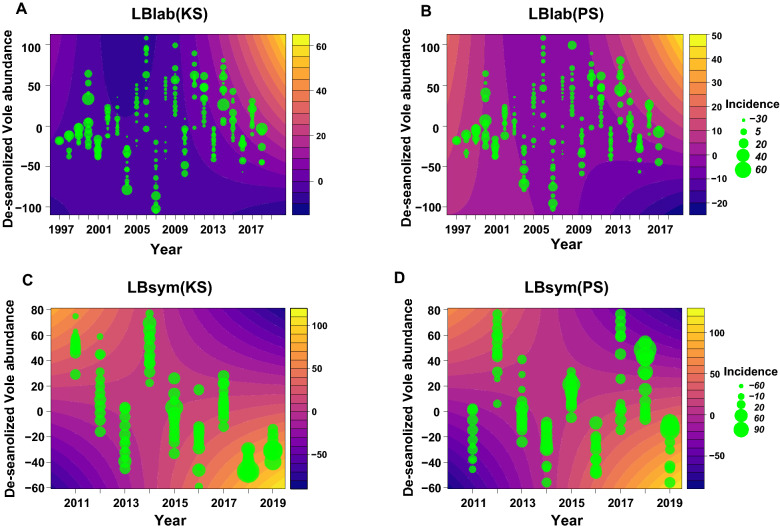
Contour plots showing the regression surfaces drawn on the grounds of fixed effects of linear mixed-effect models explaining laboratory-diagnosed LB (**A**,**B**) and symptom-diagnosed LB (**C**,**D**) incidence in relation to bank vole abundance 12 (**A**,**B**) and 10 (**C**,**D**) months earlier in two hospital districts (PS: Northern Savo, KS: Central Finland). The circles depict the data points, circle size increasing with increasing incidence of LB. Both LB incidence and bank vole abundance data were de-seasonalized by subtracting month-specific mean from each observation. The coefficients used in drawing the regression surfaces are presented in Tables 1 and 2. De-seasonalized bank vole abundance 24 months earlier was set to the mean of that variable for the derivation of the regression surfaces (bank vole abundance 24 months earlier does not affect the shape of the regression surface, only the elevation of it). Figure created by using R version 3.5.3^58^.

**Figure 3:**
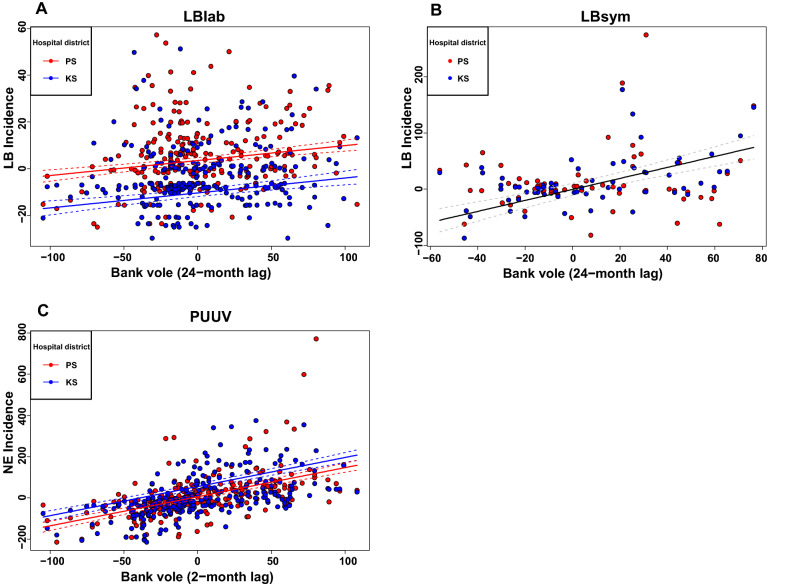
Incidence of LB laboratory (**A**), LB symptoms (**B**) and PUUV infection (**C**) in relation to bank vole abundance 24 (**A**,**B**) and 2 (**C**) months earlier. Points indicate observations from Central Finland (KS; blue symbols) and Northern Savo (PS; red symbols) hospital districts. Fitted regression lines (unbroken lines) and their 95% confidence intervals (dashed lines) based on linear mixed-effect models are also presented, separately for the two hospital districts (blue lines for Central Finland; red lines for Northern Savo) when there was a hospital district effect (**A**,**C**) and for pooled data (black line) when hospital district effect was not found (**B**). All the time series were de-seasonalized (see text for details). The coefficients used in drawing the regression lines are presented in Tables 1 and 2. In each plot, lines are drawn for the year 2018 by keeping bank vole abundance 12 (**A**) or 10 (**B**) months earlier fixed at zero (i.e. at the month-specific mean), the slopes of the regression lines being independent of year and bank vole abundance with the 12- and 10-month lags. Figure created by using R version 3.5.3^58^.

The LMM approach revealed that the global model was superior to any other model for LBlab (Table S1). LBlab was non-linearly associated with bank vole abundance 12 months earlier, and this association changed over the years as there was an interaction between bank vole abundance with 12-month lag and each of year and year^2^ (Table 1; Fig. 2A,B). From 2001 to 2008, when the amplitude of bank vole abundance variation was high, there was a practically non-existent association between LBlab incidence and bank vole abundance 12-months earlier (Fig. 2A,B). However, the association was positive in the beginning of the time series and strongly positive in the end of it (Fig. 2A,B), when the bank vole abundance was varying less (Fig. S3). Temporal LBlab incidence variation was different in the two hospital districts (Table 1; Fig. S3A), but the association between LBlab incidence and bank vole abundance was qualitatively similar in both hospital districts for the short (Fig. 2A,B) and long time lags (Fig. 3A). LBlab incidence was positively and linearly associated with bank vole abundance 24 months earlier (Table 1; Fig. 3A).

**Table 1:** Output of linear mixed effect models (fixed effects) fitted with the maximum likelihood method explaining incidence of laboratory-diagnosed Lyme Borreliosis (LBlab) in the Central Finland (KS) and Northern Savo (PS) hospital districts from 1995 to 2019 in relation to year and bank vole abundance.

Parameter	Estimate	Standard error	*t*-value	*P*-value
Intercept	19.62	3.82	5.12	< 0.001
Year	− 3.17	0.57	− 5.55	< 0.001
Year^2^	0.10	0.019	5.38	< 0.001
Hospital district (KS)	− 13.86	2.98	− 4.64	< 0.001
Bank vole(lag12)	0.20	0.13	1.53	0.12
Bank vole(lag24)	0.063	0.016	3.91	< 0.001
Year × Hospital districts (KS)	1.042	0.20	5.14	< 0.001
Year × Bank vole(lag12)	− 0.040	0.019	− 2.089	0.037
Year^2^ × Bank vole(lag12)	0.0017	0.00066	2.66	0.0081

Month was used as a random effect. Temporal autocorrelation of residuals was modelled with the AR(2) function. Model adjusted R^2^ = 0.20.

For LBsym, there were six models within two AICc units from the best model. Hence, model averaging was conducted over this set of models (Table S2). There was an interaction between year and bank vole abundance ten months earlier (Table 2), indicating multi-annual change from a positive to a negative association between LBsym incidence and bank vole abundance ten months earlier (Fig. 2C,D). This is consistent with the high LBsym incidence in the end of the study period that coincides with low bank vole abundance (Fig. S3). LBsym incidence was not associated with bank vole abundance 24 months earlier (Table 2; Fig. 3B). There was no difference in incidence or temporal changes of incidence in LBsym between the hospital districts (Table 2).

**Table 2:** Model-averaged fixed effects (including models with ΔAICc < 2) of linear mixed-effects models explaining LB symptoms incidence from 2011 to 2019 and PUUV infection incidence from 1995 to 2019 in Central Finland (KS) and Northern Savo (PS) hospital districts.

Disease	Parameter	Averaged Estimate	Adjusted standard error	z-value	*P*-value
LBsym^a^	Intercept	− 20.88	23.75	0.87	0.37
	Year	5.79	4.31	1.34	0.17
	Hospital district(KS)	11.89	23.98	0.49	0.62
	Bank vole(lag10)	1.046	0.46	2.51	0.024
	Bank vole(lag24)	0.20	0.23	0.88	0.37
	Year × Hospital districts (KS)	− 2.19	4.46	0.49	0.62
	Year × Bank vole(lag10)	− 0.25	0.086	0.88	0.0026
PUUV^b^	Intercept	− 0.22	7.83	0.029	0.97
	Year	0.26	0.55	0.48	0.62
	Hospital district (KS)	49.29	9.18	5.36	< 0.001
	Bank vole(lag2)	1.3	0.23	5.57	< 0.001
	Year × Hospital districts (KS)	− 3.57	0.65	5.47	< 0.001
	Year × Bank vole(lag12)	0.0055	0.015	0.35	0.72

The models were fitted with the maximum likelihood method.

^a^Month was used as a random effect. Temporal autocorrelation of residuals was modelled with the AR(2) function.

^b^Month was used as a random effect. Temporal autocorrelation of residuals was modelled with the ARMA(1,1) function.

Two models were within two AICc units from the best model in explaining PUUV incidence variation (Table S3), so model averaging was used over this set of models. PUUV incidence was positively and linearly associated with bank vole abundance two months earlier (Table 2; Fig. 3C). PUUV incidence was higher in the Central Finland than the Northern Savo hospital district, temporal changes also differing between the hospital districts.

## Discussion

Rodent host abundance fluctuations are known to drive human infection epidemics, caused for example by Orthohantaviruses, which are transmitted via aerosols without a vector from rodent reservoir hosts to humans^15,20,21,29,30,68–72^. Here, we show that rodent host population abundance fluctuations are also associated with the epidemiology of rodent-associated tick-transmitted diseases in humans. The incidence of LB infections follows the bank vole abundance variations with 12- (or 10-) and 24-month time lags in Finland, which contrasts with the very short time lag of two months in the directly transmitted PUUV infection. The 12 or 10-month time lag from bank vole population changes to human LB incidence changes is in accordance with earlier work in Central Europe^39,40^ and with the hypothesis that rodent population dynamics affects *Borrelia* spp. infection prevalence in nymphal ticks and, consequently, *Borrelia* spp. infection risk in humans^33–35,73,74^. However, our results differ from those of earlier studies in two important aspects. First, the relationship between laboratory-diagnosed LB incidence (LBlab) and bank vole abundance 12 months earlier changed over the study period, showing positive association in the early and late study years, whereas in the middle of the time series the association seemed to disappear. In symptoms-diagnosed LB, the association with human incidence and bank vole abundance 10 months earlier changed from positive to negative across the studied years. In contrast, Tkadlec et al.^39^ reported a (static) positive cross-correlation between vole abundance (12-month lag) and tick-borne disease incidence. Moreover, Bogdziewicz et al.^39^ found a positive (non-significant) association between Google search volume for mice in year *t* and Google search volume for tick in year *t* + 1, also suggesting approximately 12-month delay from rodent population peaks to high incidence of tick-borne diseases. Second, we found a positive linear association between laboratory-diagnosed human LB incidence and bank vole abundance 24 months earlier, while such a long time lag was not detected in Central Europe^39^. More complex rodent population dynamics, with varying strength of cyclicity, in Northern than in Central Europe^26^ is a likely explanation for these differences.

The 12- (or 10-) month lag between bank vole abundance and LB incidence likely arises because it often takes about one year for an *I. ricinus* larva that has acquired *Borrelia* spp. from infected rodent to develop into an infected nymph, which can then transmit the infection further, for instance to a human^56,75^. In addition, we found that the abundance of bank voles 24 months earlier is positively associated with the current human LB incidence (Fig. 3A,B). A 24-month delay between bank vole abundance and LB infection incidence may be a consequence of human infections caused by adult female ticks, rather than nymphs as the development from a larva to adult generally takes two years. While nymphs are regarded as most relevant in transmitting pathogens to humans, due to their small size and high abundance^16,76^, infected adult female ticks may also transmit *Borrelia* infections^77,78^. This is especially the case for *I. persulcatus*, which typically bites humans only as an adult^79–82^. Interestingly, both *I. ricinus* and *I. persulcatus* are commonly found in the two hospital districts included in the current study^83^. The incidence of *Borrelia* spp. infection is also higher in *I. persulcatus* than in *I. ricinus* adults^28,82,84^. Hence, humans are predominantly infected by *I. ricinus* nymphs and *I. persulcatus* adults^80,81,84^, which is likely to explain our result showing both approximately 12-month as well as 24-month time lags between rodent abundance and human LB incidence.

In our study site, bank vole abundance varied predominantly seasonally in the early years (1995–2002), whereas multiannual cycles with high amplitude and prolonged low phase for over a year (*i.e.*, the classical vole cycles^21,85,86^;) emerged in subsequent years, until the amplitude of cycles dampened towards the end of the time series (Fig. S3). Both the dependency of human LBlab incidence on bank vole abundance 12 months earlier and the dependency of human LBsym incidence and bank vole abundance 10 months earlier changed along with changing bank vole population dynamics (Fig. 2). In the early years (before 2000), the human LBlab incidence was positively associated with bank vole abundance 12 months earlier. However, between 2003 and 2011, when cycles dominated bank vole population dynamics, LBlab incidence was practically independent of bank vole abundance 12 months earlier. This may be because the strong bank vole population abundance variations are not favorable for the maintenance of *Borrelia* spp. in the vole population. First, the bank vole population abundance may be too low to support larval ticks, which must feed on alternative hosts that may not be competent for the *Borrelia* spp. pathogen and thus do not support the transmission of the pathogen from infected ticks to uninfected ones^87,88^. Second, during increase and peak phases of the vole population cycle*,* high availability of rodent hosts may decrease the likelihood that an infected nymph and a susceptible larva feed on the same rodent host, which may, in turn, decrease *Borrelia* spp. infection prevalence in the bank vole population. Consequently, the pathogen prevalence in ticks (nymphs and adults) may decrease, leading to a lower infection risk in humans^89,90^. Hence, the circulation of the tick-borne pathogens may not be maintained in cyclically varying vole populations. The maintenance of *Borrelia* spp. in a host community may then rely on alternative non-vole hosts, which would make human LB incidence independent of vole population abundance. Tick larvae may use alternative hosts which would reduce *Borrelia* spp. infection in ticks and so hinder transmission of *Borrelia* spp. to humans^32,91,92^. Indeed, the abundance of infected nymphs has been associated with rodent abundance changes, but with a delay of approximately one year^32,74,89,90^. After 2011, when the amplitude of bank vole population fluctuations dampened, LBlab incidence become again positively associated with bank vole abundance 12 months earlier, while the association between LBsym incidence and bank vole abundance 10 months earlier changed from positive to negative. This difference between LBlab and LBsym could be explained, for example, if different *Borrelia* species typically cause laboratory- and symptoms-based diagnoses^93^ and the transmission of these *Borrelia* species is differently associated with bank vole abundance. Moreover, the LBsym time series starts only after the classical vole cycles had dampened in the bank vole population (Fig. S3), and because this change in bank vole population dynamics appeared important for the dependency of LBlab on bank vole abundance, it necessarily means that it is not possible to capture the same effect with the LBsym data even if there would be similar associations with bank vole abundance for the two LB data sets.

Symptom-diagnosed LB follows bank vole abundance with shorter time lags than laboratory-diagnosed LB. This is because LBsym, which includes predominantly erythema migrans rash, typically appear soon after (3–30 days) an infectious tick bite and, thus, leads to diagnoses soon after the infection. However, not all infections cause erythema rash but unspecific symptoms that may be diagnosed with laboratory tests. Hence, typically laboratory-diagnosed with a longer time lag since the tick-bite. In addition, antibodies may become detectable only longer time period from the infection (earliest 3–5 weeks from the tick bite), which also delay the diagnostics. Temporal changes in LBlab incidence were not completely parallel in the two hospital districts. A potential explanation could be variation in population dynamics of bank voles or tick vectors between the hospital districts, but lack of data precludes a rigorous assessment of this. Although the two studied hospital districts are close to each other, numbers of diagnosed cases may be affected by differences in diagnostic awareness. There was not any clear evidence of a temporal increase in LB incidence in the studied hospital districts, contrary to what has been found in Finland as a whole^44^.

Infection dynamics of PUUV are less complex than that of LB, because PUUV is directly transmitted from bank voles to humans without vectors. The short time lag with which PUUV infection incidence follows bank vole abundance is a consequence of the two to six weeks incubation period for the development of PUUV symptoms^28,94^. Hence, both PUUV incidence and bank vole abundance show similar 3-year cycles (see Figs S4 and S5), which is in accordance with earlier studies^21,26,95,96^. Because we could find the known dependency of human PUUV incidence on bank vole abundance two months earlier^21^ with the same data that were used in analyzing LB epidemiology, our data appear informative of potential associations between LB incidence and bank vole abundance too.

To conclude, our results support the previous findings that the rodent host population dynamics appears important for the epidemiology of rodent-associated orthohantavirus^21,27–30^ as well as *Borrelia burgdorferi* sensu lato infections in humans^31,33,35–37^ also in the northernmost zone of the pathogen distribution range in Northern Europe. While vectors induce time lags in pathogen transmission, the circulation of tick-borne pathogens and the human disease epidemics are potentially complicated by the presence of alternative hosts for the pathogens and vectors as well as environmental conditions that affect the vectors and the hosts. Therefore, a comprehensive understanding of the epidemics of rodent-associated tick-borne zoonoses, like Lyme Borreliosis, requires a community-level analysis where the roles of alternative hosts, environmental conditions and their interactions with vectors are assessed. Yet, our study clearly highlights the epidemiological importance of the population dynamics of rodent reservoir dynamics.

## Supplementary Information

Supplementary Information.
