# Supplementary material for: Rodent host population dynamics drive zoonotic Lyme Borreliosis and Orthohantavirus infections in humans in Northern Europe
Source: Sci Rep. 2021 Aug 9;11:16128. doi: 10.1038/s41598-021-95000-y (PMC8352996; doi:10.1038/s41598-021-95000-y)
Supplement: Supplementary file 1 — Supplementary Information. [file 41598_2021_95000_MOESM1_ESM.docx]

**Supplementary Information for**

Rodent host population dynamics drive zoonotic Lyme Borreliosis and Orthohantavirus infections in humans in Northern Europe

**Mahdi Aminikhah^a^*, Jukka T. Forsman^b^, Esa Koskela^c^, Tapio Mappes^c^, Jussi Sane^d^, Jukka Ollgren^d^ , Sami M. Kivelä^a^ and Eva R. Kallio^c^***

* Corresponding authors: Mahdi Aminikhah and Eva R. Kallio

Email: [mahdi.aminikhah@oulu.fi](mailto:mahdi.aminikhah@oulu.fi); eva.r.kallio@jyu.fi

**This PDF file includes:**

Tables A1 to A3

Figures S1 to S5

Tables S1 to S3

**Supplementary Appendix A: An assessment of potential weather effects on the results**

**Weather data**

We considered the North Atlantic Oscillation (NAO) index values (<https://www.ncdc.noaa.gov/teleconnections/nao>; Fig. S3) as general proxies for weather conditions relevant to our study system^1^ as fluctuations in NAO are associated with changes in temperature and precipitation in the Northern Hemisphere^2^. We used the normalized monthly data format from National Centers for Environmental Information (NOAA) on monthly NAO index.

**Statistical Analysis**

First, we removed the seasonal effect from the monthly NAO by using equation 2 presented in the main text. We wanted to take into account a potential confounding effect of weather-dependency of tick activity on the results as tick activity is known to depend on weather conditions^3–5^. Thus, we added de-seasonalized NAO index (i.e. an index of large-scale weather variation) two months earlier into the global model to see if it would explain LB incidence variation in the human population not explained by bank vole abundance variation. We considered only two-month NAO lag because tick activity in the months preceding a diagnosis is expected to have the strongest influence on LB incidence. The survival and lifespan of PUUV in the environment may be weather-dependent^6,7^, which is why we used a two-month NAO lag also when analyzing variation in PUUV infections. On the other hand, bank vole population density is known to be associated with NAO index with longer time lags^8,9^. Thus, adding long time lags of NAO could confound our inferences concerning the association between bank vole abundance and LB incidence in the human population.

**Results**

The de-seasonalized two-month NAO index (two-month lag) did not explain LBlab incidence variation (Table A1), so we ignored NAO in multi-model inference presented in the main text. For LBsym, de-seasonalized NAO index (two-month lag) did not explain variation in incidence either (Table A2), which is why NAO was ignored in multi-model inference presented in the main text.

De-seasonalized NAO index (two-month lag) did not explain PUUV infection incidence variation (Table S3). Therefore, NAO was ignored in multi-model inference concerning PUUV infection incidence variation presented in the main text.

**Table A1. Output of linear mixed effect models (fixed effects) fitted with the maximum likelihood method explaining LB laboratory incidence in Central Finland (KS) and Northern Savo (PS) hospital districts from 1995 to 2019. The de-seasonalized NAO index two months earlier was included as a fixed effect here.**

| Parameter | Estimate | Standard error | t-value | P-value |
| --- | --- | --- | --- | --- |
| Intercept | 20.17 | 3.78 | 5.32 | <0.001 |
| Year | -3.28 | 0.56 | -5.79 | <0.001 |
| Year2 | 0.11 | 0.019 | 5.65 | <0.001 |
| Hospital district(KS) | -13.89 | 2.92 | -4.75 | <0.001 |
| Bank vole(lag12) | 0.23 | 0.13 | 1.72 | 0.084 |
| Bank vole(lag24) | 0.061 | 0.016 | 3.74 | <0.001 |
| NAO(lag2) | -1.044 | 0.59 | -1.75 | 0.079 |
| Year × Hospital districts (KS) | 1.046 | 0.19 | 5.26 | <0.001 |
| Year × Bank vole(lag12) | -0.044 | 0.019 | -2.28 | 0.022 |
| Year^2^ × Bank vole(lag12) | 0.0019 | 0.00067 | 2.84 | 0.0047 |

**Month was used as random effect nested in 25 years. AR(2) correlation function was used for modelling temporal autocorrelation of residuals. Model-adjusted R^2^=0.24.**

**Table A2. Output of linear mixed effect models (fixed effects) fitted with the maximum likelihood method explaining LB symptoms incidence in Central Finland (KS) and Northern Savo (PS) hospital districts from 2011 to 2019. The de-seasonalized NAO index two months earlier was included as a fixed effect here.**

| Parameter | Estimate | Standard error | t.value | P-value |
| --- | --- | --- | --- | --- |
| Intercept | -46.52 | 23.16 | -2.0082 | 0.047 |
| Year | 10.28 | 4.24 | 2.42 | <0.001 |
| Hospital district(KS) | 45.59 | 26.0095 | 1.75 | 0.0821 |
| Bank vole(lag10) | 1.16 | 0.44 | 2.60 | <0.001 |
| Bank vole(lag24) | 0.36 | 0.20 | 1.76 | 0.080 |
| NAO(lag2) | 5.44 | 4.36 | 1.24 | 0.21 |
| Year × Hospital districts (KS) | -8.57 | 4.81 | -1.77 | 0.077 |
| Year × Bank vole(lag10) | -0.25 | 0.085 | -3.0051 | <0.001 |

**Month was used as random effect nested in 9 years. AR(2) correlation function was used for modelling temporal autocorrelation of residuals. Model-adjusted R^2^=0.24.**

**Table A3. Output of linear mixed effect models (fixed effects) fitted with the maximum likelihood method explaining PUUV incidence in Central Finland (KS) and Northern Savo (PS) hospital districts from 1995 to 2019. The de-seasonalized NAO index two months earlier was included as a fixed effect here.**

| Parameter | Estimate | Standard error | t.value | P-value |
| --- | --- | --- | --- | --- |
| Intercept | 0.32 | 7.93 | 0.041 | 0.96 |
| Year | 0.21 | 0.56 | 0.38 | 0.69 |
| Hospital districts (KS) | 48.77 | 9.13 | 5.33 | <0.001 |
| Bank vole(lag2) | 1.19 | 0.32 | 3.63 | <0.001 |
| NAO(lag2) | 8.25 | 4.99 | 1.65 | 0.098 |
| Year × Hospital districts (KS) | -3.53 | 0.64 | 5.44 | <0.001 |
| Year × Bank vole(lag2) | 0.016 | 0.022 | 0.74 | 0.45 |

**Month was used as random effect nested in 25 years. ARMA(1,1) correlation function was used for modelling temporal autocorrelation of residuals. Model-adjusted R^2^=0.53.**

**References**

1. Stenseth, N. C. & Mysterud, A. Weather packages: finding the right scale and composition of climate in ecology. *J. Anim. Ecol.* **74**, 1195–1198 (2005).

2. Hurrell, J. W. Decadal trends in the North Atlantic oscillation: Regional temperatures and precipitation. *Science (80-. ).* **269**, 676–679 (1995).

3. Ruiz-Fons, F., Fernández-de-Mera, I. G., Acevedo, P., Gortázar, C. & de la Fuente, J. Factors driving the abundance of Ixodes ricinus ticks and the prevalence of zoonotic I. ricinus-borne pathogens in natural foci. *Appl. Environ. Microbiol.* **78**, 2669–2676 (2012).

4. Randolph, S. E., Green, R. M., Peacey, M. F. & Rogers, D. J. Seasonal synchrony: The key to tick-borne encephalitis foci identified by satellite data. *Parasitology* **121**, 15–23 (2000).

5. Randolph, S. E. & Rogers, D. J. Fragile transmission cycles of tick-borne encephalitis virus may be disrupted by predicted climate change. *Proc. R. Soc. B Biol. Sci.* **267**, 1741–1744 (2000).

6. Tersago, K. *et al.* Hantavirus disease (nephropathia epidemica) in Belgium: Effects of tree seed production and climate. *Epidemiol. Infect.* **137**, 250–256 (2009).

7. Linard, C., Tersago, K., Leirs, H. & Lambin, E. F. Environmental conditions and Puumala virus transmission in Belgium. *Int. J. Health Geogr.* **6**, 55 (2007).

8. Šipoš, J., Suchomel, J., Purchart, L. & Kindlmann, P. Main determinants of rodent population fluctuations in managed Central European temperate lowland forests. *Mammal Res.* **62**, 283–295 (2017).

9. Palo, T. Time Series Analysis Performed on Nephropathia Epidemica in Humans of Northern Sweden in Relation to Bank Vole Population Dynamic and the NAO Index. *Zoonoses Public Health* **56**, 150–156 (2009).


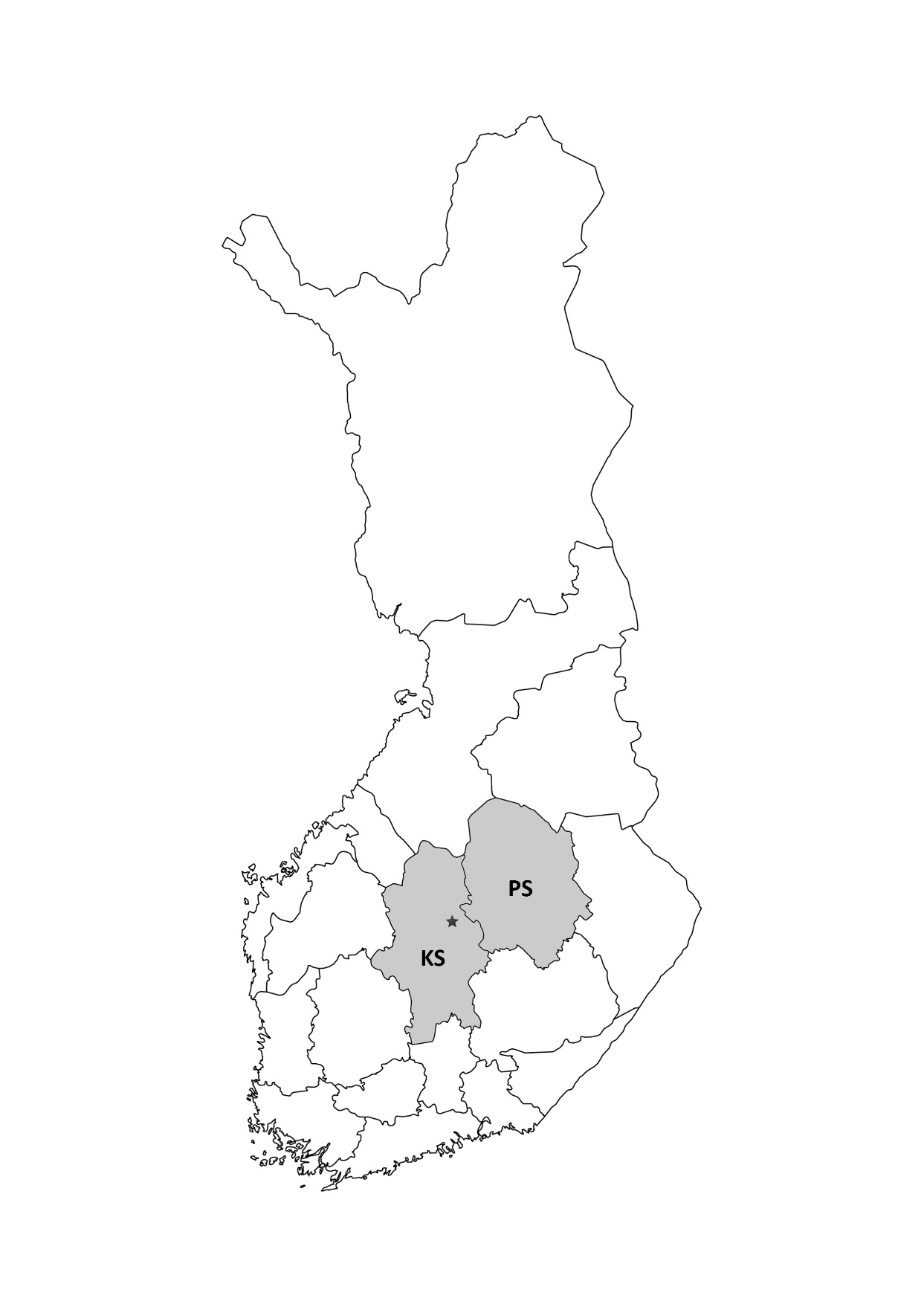


**Figure S1.** Map of Finland, showing Central Finland (KS) and Northern Savo (PS) hospital districts where the human cases of Lyme borreliosis (LB; symptom-diagnosed and laboratory-diagnosed) and PUUV infection cases were collected (grey), and the bank vole population study site in Konnevesi (star). The map was created by using “sp”^1,2^ package in R version 3.5.3^3^.


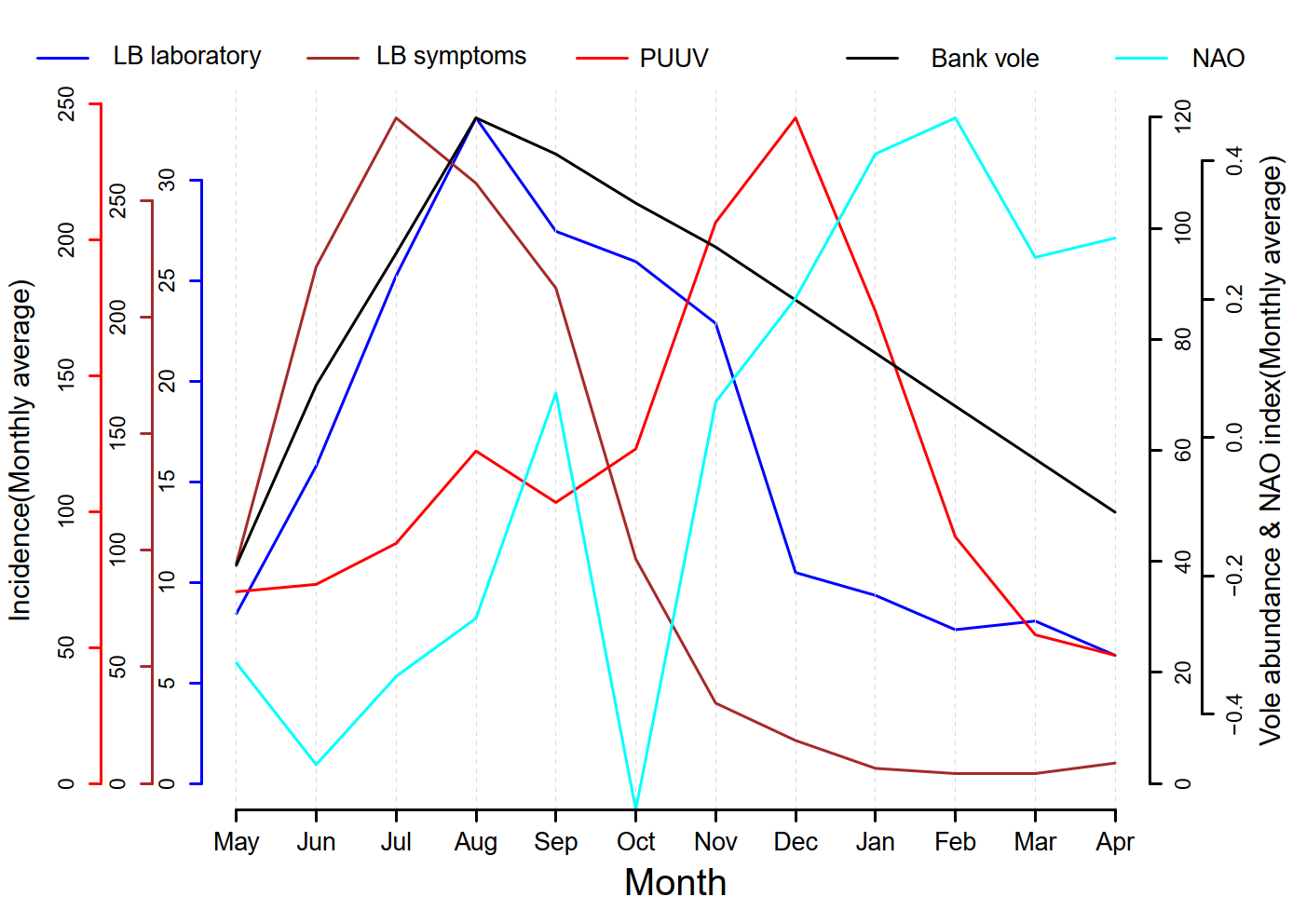


**Figure S2.** Monthly average (e.g., mean of May in all years) of LB laboratory, LB symptoms and PUUV incidence in Central Finland (KS) and Northern Savo (PS) hospital districts (hospital districts combined for calculating the monthly means) as well as bank vole abundance index (number of caught individuals per 160 trapping nights) and NAO index in 1995-2019. Figure created by using R version 3.5.3^3^.


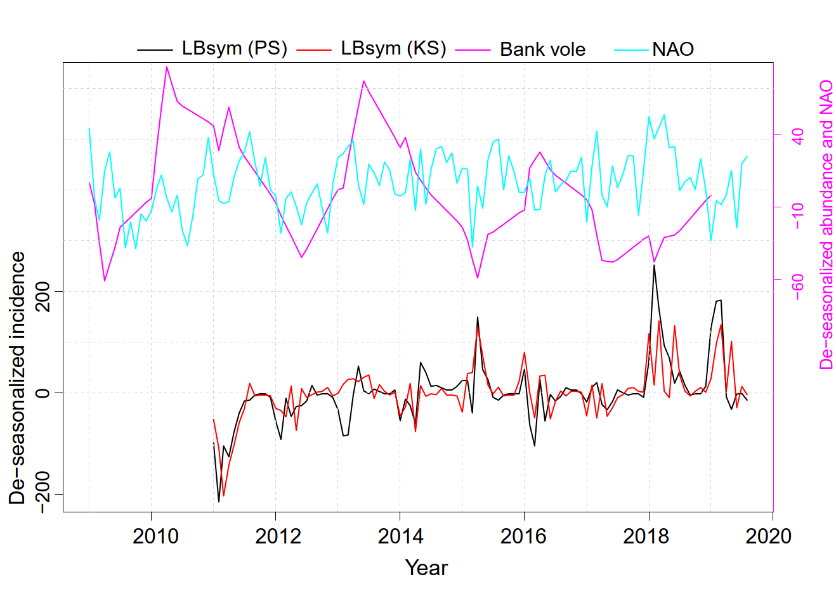

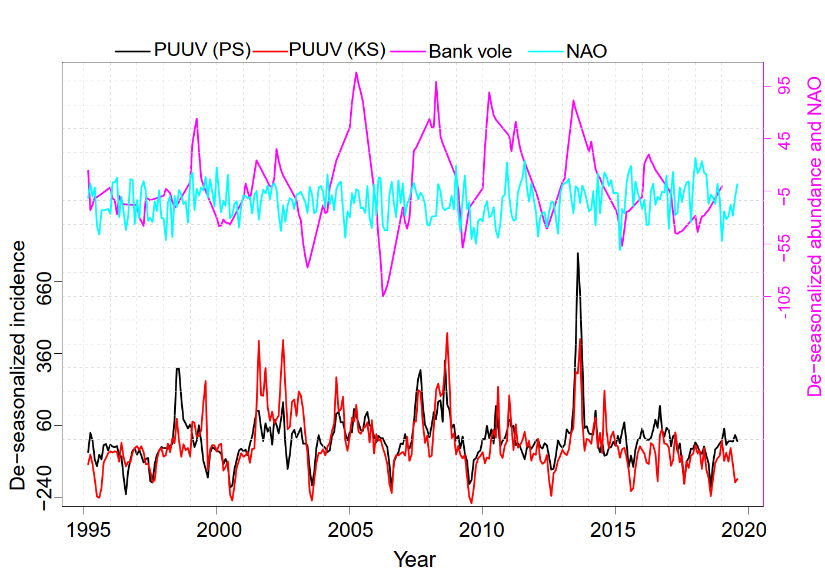

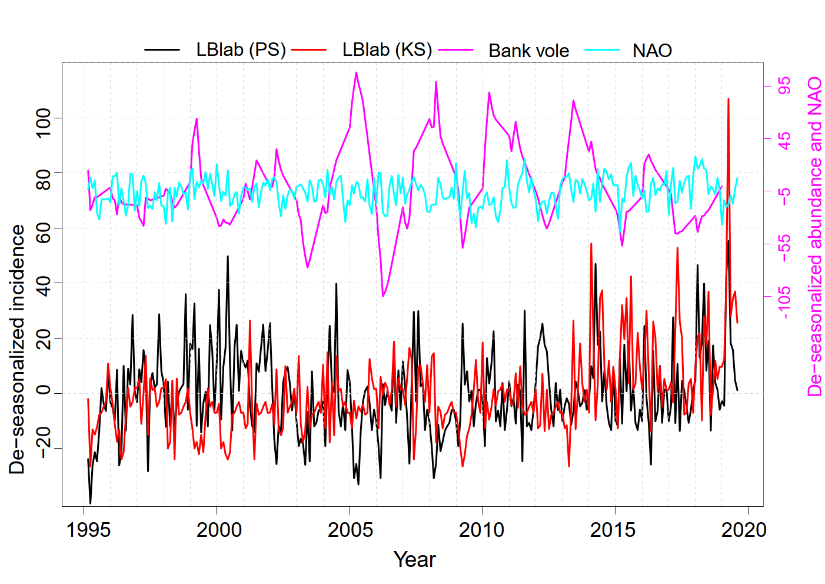


C

B

A

**Figure S3.** De-seasonalized (see text for details) NAO index (rescaled by multiplying with 20 to increase the amplitude of fluctuations for readability) and monthly bank vole abundance index (number of caught individuals per 160 trapping nights) in biological years 1995-2019 (see text for definition of the biological year) in Central Finland, together with monthly incidence of human infections in Northern Savo (PS) and Central Finland (KS) hospital districts. The human infection data are for laboratory-diagnosed Lyme Borreliosis (LB) cases in 1995-2019 (**A)**, symptom-diagnosed LB cases in 2011-2019 (**B**) and PUUV infection incidence in in 1995-2019 (**C**). Note that the bank vole and NAO data are the same in each panel A–C. Figure created by using “plotrix”^4^ package in R version 3.5.3^3^.


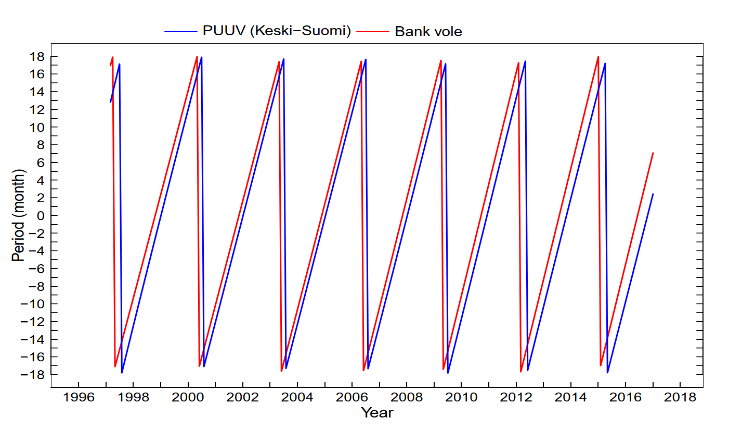

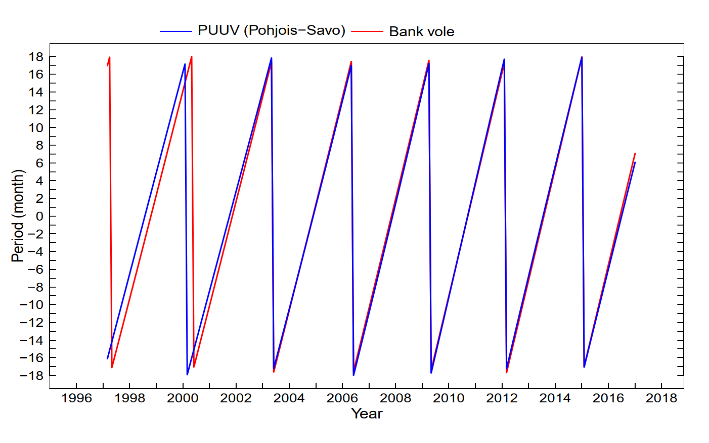

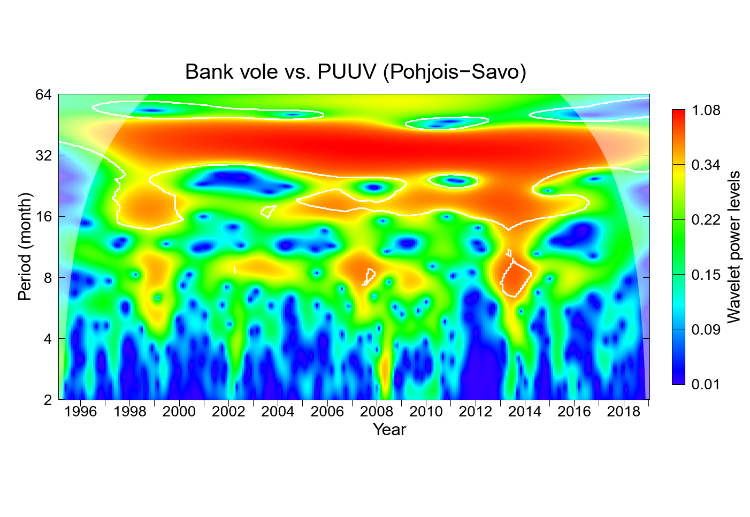


**A**

**B**


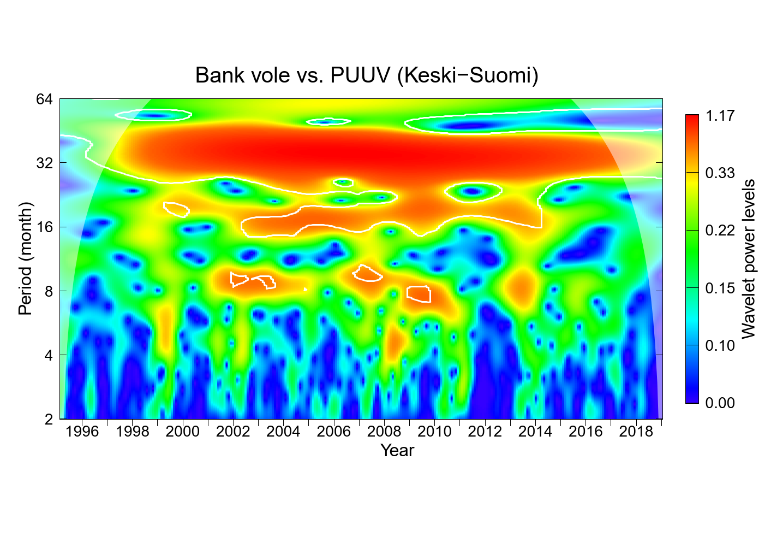


**D**

**C**

**Figure S4. Cross-wavelet power spectra and phase-difference between human infection incidence (PUUV) and bank vole abundance in two hospital distrricts.** (**A**) Cross-wavelet power between bank vole abundance and PUUV incidence in Northern Savo. (**B**) Phase-difference plot for PUUV incidence (blue line) and bank vole abundance (red line) for the periodicity with a wavelength of 36 months in Northern Savo. **(C)** Cross-wavelet power between bank vole abundance and PUUV incidence in Central Finland. **(D)** Phase-difference plot for PUUV incidence (blue line) and bank vole abundance (red line) for the periodicity with a wavelength of 36 months in Central Finland. In **A**, and **C** the coherence power is represented from low values (blue) to high values (red). Light color at edges indicates areas influenced by edge effects. All the time series are de-seasonalized (see Material and methods for more details). Figure generated using R version 3.5.3^3^ software with the ‘WaveletComp’^5^ package.


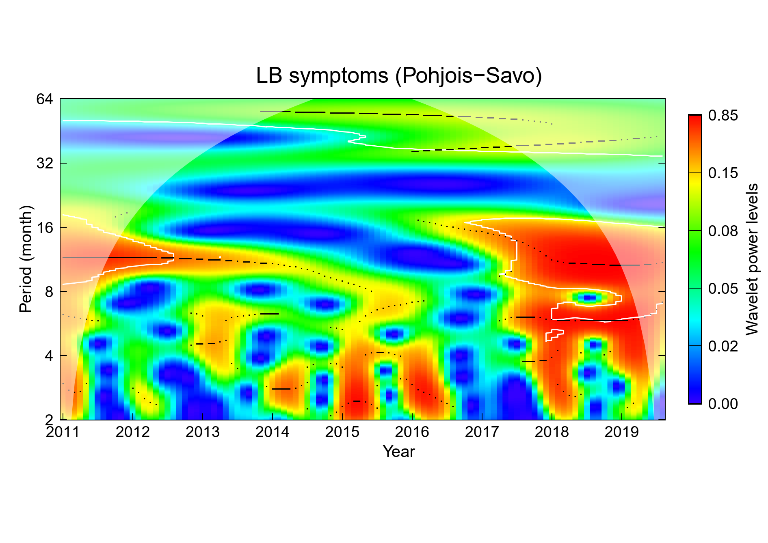

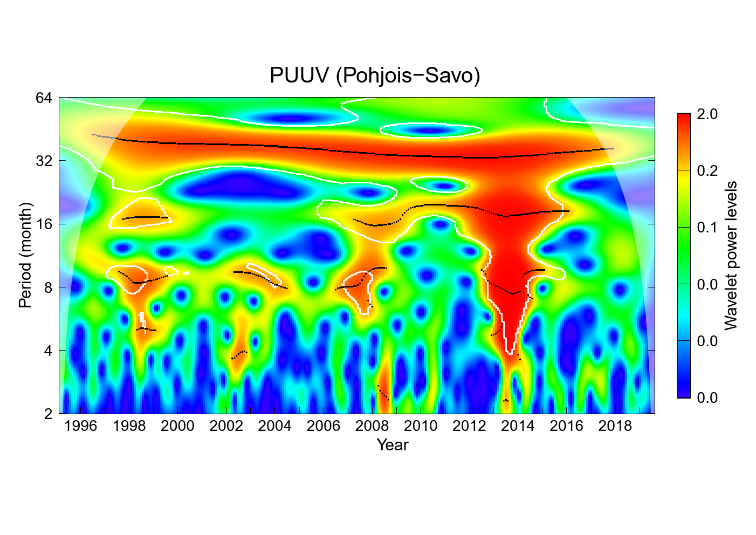

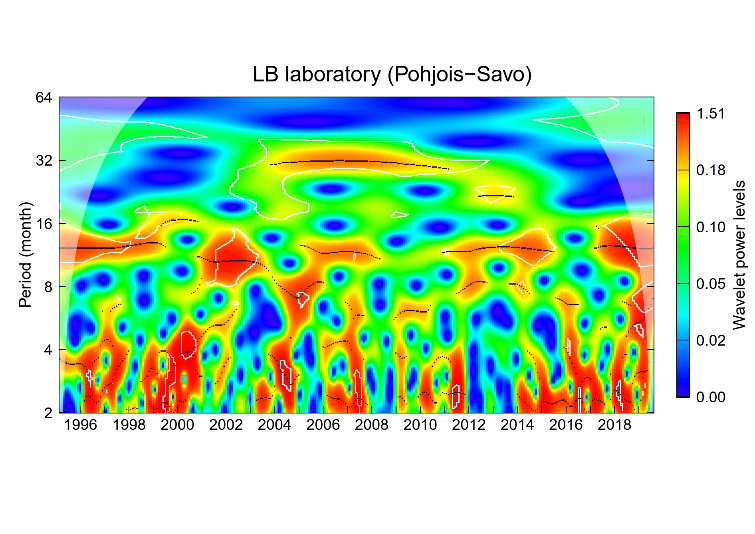


**C**

**B**

**A**

**Figure S5. Wavelet power spectrum human infection incidence A) LB laboratory, B) LB symptoms, C) PUUV in Northern Savo.** In **A**, **B**, and **C** the coherence power is represented from low values (blue) to high values (red). Light color at edges indicates areas influenced by edge effects. All the time series are de-seasonalized (see Material and methods for more details). Figure generated using R version 3.5.3^3^ software with the ‘WaveletComp’^5^ package.

**Table S1. Best models explaining laboratory diagnosed LB (LBlab) incidence in Central Finland (KS) and Northern Savo (PS) hospital districts. Models are ranked by AICc. Only models with Akaike weight >0.05 are shown. Model with lowest AICc is reported in the main document.**

| Model^a^ | df | AIC_c_ | ΔAIC_c_ | Akaike weight |
| --- | --- | --- | --- | --- |
| 1/2/3/4/5/6/7/8 | 13 | 3977.40 | 0.00 | 0.70 |
| 1/2/3/4/5/7/8 | 12 | 3979.66 | 2.26 | 0.24 |

**^a^The numbers refer to model terms as follows: 1= bank vole(lag12), 2= bank vole(lag24), 3= Hospital district (Central Finland), 4=year, 5= year^2, 6=bank vole(lag12)×year, 7= bank vole(lag12)×year^2, 8=year×Hospital district (Central Finland)**

**Table S2. Best models explaining symptoms diagnosed LB (LBsym) incidence in Central Finland (KS) and Northern Savo (PS) hospital districts. Models are ranked by AICc. Only models with Akaike weight >0.05 are shown. ΔAIC_c_ <2 was used for model averaging (Table 2 in main document).**

| Model^a^ | df | AIC_c_ | ΔAIC_c_ | Akaike weight |
| --- | --- | --- | --- | --- |
| 1/2/4/5 | 9 | 1351.76 | 0.00 | 0.33 |
| 1/4/5 | 8 | 1352.42 | 0.66 | 0.24 |
| 1/2/3/4/5/6 | 11 | 1353.24 | 1.49 | 0.16 |
| 1/3/4/5/6 | 10 | 1354.05 | 2.29 | 0.10 |
| 1/2/3/4/5 | 10 | 1354.09 | 2.33 | 0.10 |
| 1/3/4/5 | 9 | 1354.71 | 2.95 | 0.07 |

**^a^The numbers refer to model terms as follows: 1= bank vole(lag10), 2= bank vole(lag24), 3= Hospital district (Central Finland), 4=year, 5= bank vole(lag12)×year, 6= Hospital district(Central Finland) ×year**

**Table S3. Best models explaining PUUV infection incidence in Central Finland and Northern Savo hospital districts. Models are ranked by AICc. Only models with Akaike weight >0.05 are shown.** **ΔAIC_c_ <2 was used for model averaging (Table 2 in main document).**

| Model^a^ | df | AIC_c_ | ΔAIC_c_ | Akaike weight |
| --- | --- | --- | --- | --- |
| 1/2/3/5 | 9 | 5948.83 | 0.00 | 0.68 |
| 1/2/3/4/5 | 10 | 5950.36 | 1.53 | 0.32 |

**^a^The numbers refer to model terms as follows: 1= bank vole(lag2), 2= Hospital district (Central Finland) 3=year, 4= bank vole(lag2)×year, 5= Hospital district (Central Finland) ×year**

**References**

1. Pebesma, E. J. & Bivand, R. S. Classes and methods for spatial data in {R}. *R News* **5**, 9–13 (2005).

2. Bivand, R. S., Pebesma, E. J. & Gomez-Rubio, V. *Applied spatial data analysis with {R}, Second edition*. (Springer, NY, 2013).

3. R Core Team. R: A Language and Environment for Statistical Computing. *R Found. Stat. Comput. Vienna, Austria* (2019).

4. Lemon, J. Plotrix: a package in the red light district of R. *R-News* **6**, 8–12 (2006)

5. Rösch, A. & Schmidbauer, H. WaveletComp: Computational Wavelet Analysis. R package version 1.1. (2018).
